# The Ameliorative Effects of Carnosine on the In Vitro Developmental Competence of Bovine Oocytes

**DOI:** 10.3390/antiox15070828

**Published:** 2026-06-30

**Authors:** Xuan Leng, Bo-Jing Liu, Ren An, Si-Ying Chen, Kang Li, Dong Wang, Yun-Wei Pang

**Affiliations:** Institute of Animal Sciences, Chinese Academy of Agricultural Sciences, Beijing 100193, China; 82101232378@caas.cn (X.L.); 18632847220@163.com (B.-J.L.); junhuaishi@163.com (R.A.); 82101222353@caas.cn (S.-Y.C.); l2311123076@163.com (K.L.)

**Keywords:** carnosine, melatonin, bovine, oocyte, embryo, oxidative stress

## Abstract

Carnosine is a naturally occurring endogenous dipeptide with great potential to improve reproductive function and fertility. In this study, supplementation of 1 μg/mL carnosine during in vitro maturation (IVM) significantly enhanced the developmental competence and quality of the resulting bovine embryos. Carnosine treatment effectively elevated mitochondrial membrane potential, mitochondrial activity, and ATP content in oocytes. Moreover, it strengthened the antioxidant and anti-apoptotic capacities of oocytes, as evidenced by reduced intracellular reactive oxygen species (ROS) levels, lowered DNA damage and an early apoptosis rate, alongside increased glutathione (GSH) content, an elevated *BCL2*/*BAX* mRNA ratio, and upregulation of antioxidant genes *SOD1*, *CAT*, *GPx1*, and *GPx4.* Notably, combined application of 1 μg/mL carnosine during IVM and 10^−7^ M melatonin during in vitro culture (IVC) synergistically improved both blastocyst development and quality. Collectively, these findings provide novel evidence supporting the therapeutic potential of carnosine in optimizing in vitro embryo production in bovine, and highlight the value of stage-specific supplementation strategies to further improve embryonic development efficiency.

## 1. Introduction

The application of in vitro embryo production (IVEP) enables the generation of transferable embryos from well-bred donors, thereby accelerating genetic progress and improving breeding efficiency [1,2,3]. However, the outcome of IVEP remains unsatisfactory, with the average blastocyst formation rate in cattle being only 30–40% [4]. The compromised development of in vitro-generated embryos is partly attributable to the suboptimal culture conditions, which differ considerably from the physiological in vivo microenvironment. In vivo, oocytes and embryos depend on a dynamic nutrient supply from the maternal reproductive tract and a precisely regulated redox environment sustained by maternal antioxidants and enzymatic systems [5,6]. By contrast, in vitro systems entirely rely on the composition of the culture media and frequently lead to excessive accumulation of reactive oxygen species (ROS) [7], which can attack cellular constituents such as nucleic acids, lipids, and proteins, ultimately compromising embryo developmental competence and quality [8]. Consequently, optimizing in vitro conditions is essential for obtaining higher blastocyst yields and improved embryo quality.

Carnosine, the natural dipeptide of β-alanine and L-histidine, is widely distributed in various tissues and organs, exhibiting a distinct enrichment in cardiac and skeletal muscles, alongside the central nervous system [9,10]. Accumulating evidence indicates that carnosine performs diverse physiological functions, including pH buffering, antioxidant, metal chelation, and anti-inflammatory activities [11,12,13]. Owing to its well-demonstrated therapeutic potential, this dipeptide has been explored in numerous studies related to both male and female reproductive processes [12]. In male reproductive health, carnosine enhances sperm resistance during biotechnological procedures through the inhibition of oxidative insults and DNA damage, alongside the maintenance of mitochondrial and redox homeostasis [14,15]. In female reproduction, maternal supplementation of carnosine improves antioxidant capacity in mouse offspring, as reflected by reduced malondialdehyde (MDA) levels and elevated superoxide dismutase (SOD), glutathione peroxidase (GPx), and total antioxidant status (TAS) levels [16]. Moreover, the addition of carnosine to the cryopreservation solution improves the viability of slow-frozen bovine embryos by protecting the mitochondrial membrane against oxidative injury [17]. Collectively, these results indicate that carnosine holds promise for improving reproductive function and fertility. Nevertheless, its specific effects on oocyte maturation potential remains to be fully elucidated.

The present study aimed to assess the involvement of carnosine in bovine oocyte maturation and the ensuing in vitro embryonic developmental capacity, with a preliminarily inquiry into its underlying mechanisms. Importantly, based on previous studies on melatonin [18,19], we performed stage-specific supplementation with carnosine during oocyte maturation and melatonin during embryo culture to evaluate their synergistic effects. Our findings may provide a promising strategy for optimizing IVEP efficiency.

## 2. Materials and Methods

Unless otherwise indicated, chemicals and reagents were obtained from Sigma-Aldrich (St. Louis, MO, USA) or Thermo Fisher Scientific (Waltham, MA, USA). The γH2AX antibody and Alexa Fluor 488/594-conjugated secondary antibodies were obtained from Cell Signaling Technology (Beverly, MA, USA), and the CDX2 antibody was obtained from Proteintech (Rosemont, IL, USA).

### 2.1. Experimental Design

Bovine cumulus–oocyte complexes (COCs) were randomly allocated to maturation media containing various concentrations of carnosine (0, 0.5, 1, 5, or 10 μg/mL) for 22–24 h. The influences of carnosine were first evaluated on oocyte first polar body extrusion, cleavage and blastocyst formation after in vitro fertilization (IVF), as well as the average total cell number per blastocyst. Based on the optimal concentration determined, COCs were randomly divided into two experimental groups: control group and carnosine treatment group (1 μg/mL). After in vitro maturation (IVM), all viable denuded oocytes were collected for further mechanistic analyses.

To evaluate the effect of carnosine on mitochondrial function, the mitochondrial membrane potential, mitochondrial activity, and ATP content were assessed. The antioxidative ability of carnosine was examined by measuring ROS and glutathione (GSH) levels, along with the mRNA expression of antioxidant-related genes (*SOD1*, *CAT*, *GPx1*, and *GPx4*). Meanwhile, its anti-apoptotic capacity was evaluated through detection of DNA double-strand breaks (γH2AX level), early apoptosis rate, and the expression of apoptosis-related genes (*BAX* and *BCL2*).

To further improve developmental efficiency, the stage-specific combined effects of carnosine and melatonin during IVEP were determined. Four treatment groups were established: (1) Control—both maturation and culture were performed in basic media; (2) Carnosine (IVM) only—the maturation medium was supplemented with 1 μg/mL carnosine; (3) Melatonin (IVC) only—the culture medium was supplemented with 10^−7^ M melatonin; and (4) Carnosine (IVM) + Melatonin (IVC)—both media were supplemented (1 μg/mL carnosine in maturation medium and 10^−7^ M melatonin in culture medium). Embryonic development parameters, including cleavage rate, blastocyst formation rate, the average total cell number per blastocyst, and the number of inner cell mass (ICM) and trophectoderm (TE) cells, were recorded for all groups.

### 2.2. IVEP Procedure

The IVEP procedure was conducted according to our previously described protocol [20]. COCs were recovered and matured for 22–24 h in maturation medium at 38.5 °C under a humidified atmosphere containing 5% CO_2_. The basic IVM medium consisted of TCM-199 supplemented with 0.01 IU/mL follicle-stimulating hormone, 0.01 IU/mL luteinizing hormone, 1 μg/mL estradiol, 10 μg/mL heparin, 10% (*v*/*v*) fetal bovine serum (FBS), 100 U/mL penicillin, and 100 μg/mL streptomycin. After maturation, cumulus cells were partially removed by repeated gentle pipetting, and 15–20 denuded COCs were placed in each 50 μL droplet of Brackett and Oliphant (BO) fertilization medium [21] supplemented with 10 μg/mL heparin and 4 mg/mL fatty acid-free bovine serum albumin (BSA). For IVF, frozen semen was thawed at 37 °C for 30 s, washed twice by centrifugation at 1800 rpm for 8 min in 6 mL BO medium containing 10 mM caffeine and 4 mg/mL fatty acid-free BSA, and then adjusted to a final concentration of 2 × 10^6^ sperm/mL. Each fertilization droplet received 50 μL of sperm suspension, then was incubated at 38.5 °C in humidified air with 5% CO_2_ for 18 h. After fertilization, presumptive zygotes were gently pipetted to remove the remaining cumulus cells and spermatozoa, and 25–30 zygotes were cultured in each 100 μL droplet of CR1aa medium [22] supplemented with 6 mg/mL fatty acid-free BSA. On day 2 post fertilization, cleaved embryos were selected and transferred to CR1aa medium containing 10% (*v*/*v*) FBS for an additional 5-day culture, with half of the medium refreshed every 2 days. The cleavage and blastocyst rates were recorded at day 2 and 7 post fertilization, respectively.

### 2.3. Mitochondrial Function Assessment

Mitochondrial activity and mitochondrial membrane potential were assessed using MitoTracker™ Red CMXRos dye (Molecular Probes, Eugene, OR, USA) and the MitoProbe™ JC-1 assay kit (Life Technologies Inc, Grand Island, NY, USA), respectively, following procedures mentioned in our previously report [23].

For mitochondrial activity analysis, denuded oocytes (replicates = 4, oocytes, *n*; control, *n* = 42; carnosine, *n* = 40) were fixed in PBS containing 4% (*w*/*v*) paraformaldehyde (PFA) at 37 °C for 15 min. After fixation and rinsing, oocytes were stained with 100 nM MitoTracker™ Red CMXRos for 30 min at 37 °C in the dark. After another three washes, samples were counterstained with 2 μg/mL 4′,6-diamidine-2′-phenylindole (DAPI), mounted on glass slides, and immediately visualized under a laser confocal microscope (TCS SP8, Leica, Wetzlar, Germany). Mean fluorescence intensity was quantified using Image J 1.54p software (National Institutes of Health, Bethesda, MD, USA).

For mitochondrial membrane potential measurement, denuded oocytes (replicates = 4, oocytes, *n*; control, *n* = 38; carnosine, *n* = 37) were washed three times in 0.1% PVA/PBS and stained with 4 μM JC-1 solution for 30 min at 38.5 °C in the dark. After staining, oocytes were rinsed, mounted, and imaged under a laser confocal microscope. Mitochondrial membrane potential was expressed as the red/green fluorescence intensity ratio calculated with Image J 1.54p software.

Intracellular ATP content was examined using the BODIPY™ FL ATP (Thermo Fisher Scientific, Waltham, MA, USA) assay according to the manufacturer’s protocol. Denuded oocytes (replicates = 4, oocytes, *n*; control, *n* = 57; carnosine, *n* = 44) were fixed and permeabilized with 4% PFA containing 0.5% (*v*/*v*) Triton X-100 at 37 °C for 30 min. The fixed and permeabilized oocytes were rinsed in 0.1% PVA/PBS, then incubated with 5 mM BODIPY™ FL ATP for 30 min at 38.5 °C in the dark. After the removal of excess probe, nuclei were counterstained with 2 μg/mL DAPI before mounting. Fluorescence signals were examined under a laser confocal microscope (TCS SP8, Leica, Wetzlar, Germany).

### 2.4. Determination of Intracellular ROS and GSH Levels

Intracellular ROS (replicates = 4, denuded oocytes, *n*; control, *n* = 44; carnosine, *n* = 40) and GSH (replicates = 4, denuded oocytes, *n*; control, *n* = 58; carnosine, *n* = 58) levels were measured using 2′,7′-dichlorodihydrofluorescein diacetate (DCHFDA) and ThiolTracker™ Violet GSH detection reagent (Invitrogen/Molecular Probes, Carlsbad, CA, USA), respectively, in accordance with the manufacturers’ instructions. For each group, denuded oocytes were exposed to 100 μM DCHFDA or 20 μM ThiolTracker™ Violet solution for 30 min at 37 °C in the dark. Unbound dye was removed by three rinses in 0.1% PVA/DPBS, and fluorescence images were captured using a Nikon fluorescence microscope (Nikon, Tokyo, Japan). The fluorescence intensities were analyzed using Image J 1.54p software.

### 2.5. Detection of Apoptosis-Related Indicators

DNA damage was evaluated by the immunofluorescence intensity of γH2AX. Briefly, denuded oocytes (replicates = 4, oocytes, *n*; control, *n* = 41; carnosine, *n* = 47) were fixed/permeabilized in 4% PFA containing 0.5% Triton X-100 for 45 min at 37 °C. After rinsing in 0.1% PVA/PBS, nonspecific binding was blocked overnight at 4 °C with 0.1% (*w*/*v*) BSA/PBS. Then, samples were incubated overnight at 4 °C with the anti-γH2AX antibody (1:100). After primary antibody incubation, samples were rinsed in washing buffer and labeled for 1 h at room temperature in the dark with Alexa Fluor 594-conjugated goat anti-rabbit IgG (1:200). Nuclei were stained with DAPI prior to mounting, and images were acquired using a Leica TCS SP8 confocal microscope.

Early apoptosis in oocytes (replicates = 4, denuded oocytes, *n*; control, *n* = 45; carnosine, *n* = 50) was detected using an Annexin V-FITC/dead cell apoptosis kit (Life Technologies Inc, Grand Island, NY, USA). According to the manufacture’s instruction, oocytes were first equilibrated in 1× Annexin-binding buffer for 5 min, then incubated for 15 min at room temperature in the dark in 490 μL binding buffer containing 10 μL Annexin V-FITC conjugate. After three more washes with 0.1% PVA/PBS, the oocytes were mounted on slides and analyzed under a laser confocal microscope (TCS SP8, Leica, Wetzlar, Germany).

### 2.6. Quantitative Real-Time Polymerase Chain Reaction (qPCR)

Total RNA was extracted from a pool of 100 denuded oocytes per group in each biological replicate using the RNA Easy Fast Cell Kit (DP420, Tiangen Biotech, Beijing, China). Briefly, oocytes were lysed in 350 μL Buffer RLA with 10 μL Proteinase K, incubated at room temperature for 5 min, followed by centrifugation at 12,000 rpm for 2–5 min. The supernatant was transferred to a genomic DNA removal column and centrifuged at 12,000 rpm for 30 s. The flow-through was mixed with 70% ethanol in a 1:1 ratio (*v*/*v*), loaded onto an RNase-Free spin column CR4, and washed with 700 μL Buffer RW3. On-column DNase I digestion was carried out by adding 80 μL of working solution (10 μL stock + 70 μL RDD) and incubating for 15 min at room temperature, after which another wash with 350 μL Buffer RW3 was applied. The column was then washed twice with 500 μL Buffer RW (with ethanol), air-dried, and RNA was eluted in 30 μL RNase-free ddH_2_O. The purified RNA was then reverse-transcribed using the All-in-One First-Strand Synthesis MasterMix (with dsDNase) (EG15133S, Yugong Biotech, Jiangsu, China) in a one-step protocol. The 20 μL reaction mixture contained 1 μL RNA sample, 4 μL MasterMix, 1 μL dsDNase, and 14 μL nuclease-free water. The mixture was incubated at 37 °C for 2 min, 55 °C for 15 min, and 85 °C for 5 min. The resulting cDNA was stored at −20 °C until use.

qPCR was carried out in a 10 μL reaction mixture consisting of 1 μL cDNA, 5 μL 2× Power SYBR Green PCR Master Mix, 0.2 μL each of forward and reverse primers (Table 1), and 3.6 μL nuclease-free water. The amplification program consisted of initial denaturation at 95 °C for 2 min, followed by 43 cycles of 95 °C for 15 s, 59 °C for 15 s, and 72 °C for 15 s. A melting curve analysis was then carried out from 65 °C to 95 °C, with fluorescence acquisition starting at 65 °C and measurements obtained every 15 s. A final cooling step to 4 °C was included. Each reaction was performed in triplicate. β-actin was used as an internal control, and relative gene expression was calculated using the 2^−ΔΔCt^ method. Three replicate experiments were performed per treatment.

### 2.7. Immunofluorescence Staining of ICM and TE

Blastocysts (replicates = 3, blastocysts, *n*; control, *n* = 32; carnosine (IVM), *n* = 31; melatonin (IVC), *n* = 32; carnosine (IVM) + melatonin (IVC), *n* = 33) were fixed/permeabilized in 4% PFA containing 0.5% Triton X-100 for 45 min at 37 °C. After rinsing, the samples were blocked overnight at 4 °C in 0.1% BSA/PBS. Then, blastocyst samples were incubated with anti-SOX2 (1:100) and anti-CDX2 (1:500) antibodies at 4 °C overnight. After washes, samples were labeled for 1 h at room temperature in the dark with Alexa Fluor 488-conjugated goat anti-mouse IgG (1:200) or Alexa Fluor 594-conjugated goat anti-rabbit IgG (1:200). Nuclei were stained with DAPI, and images were acquired under a Leica TCS SP8 confocal microscope.

### 2.8. Statistical Analysis

All experiments were conducted with at least three independent biological replicates unless otherwise stated. The results were expressed as means ± SEM. Graphs were generated using GraphPad Prism 8. Statistical differences were determined by one-way ANOVA, followed by Duncan’s multiple range test using the Statistical Product and Service Solutions software 26.0 (SPSS, IBM Corp., Armonk, NY, USA). *p* < 0.05 was considered statistically significant.

## 3. Results

### 3.1. Supplementation of Carnosine During IVM Enhances the Development and Quality of the Resulting Bovine Embryos

**Figure 1:**
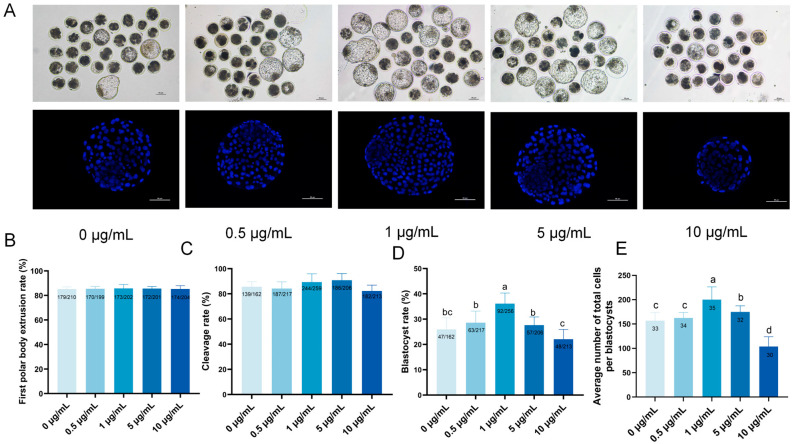
The effect of carnosine on bovine oocyte and preimplantation embryo developmental ability. (**A**) Representative images of bovine day 7 embryos and DNA staining by DAPI (blue) for each experimental group. Scale bar = 50 μm. (**B**) First polar body extrusion rate (replicates = 4, MII oocytes/COCs; 0 μg/mL, 179/210; 0.5 μg/mL, 170/199; 1 μg/mL, 173/202; 5 μg/mL, 172/201; 10 μg/mL, 174/204). (**C**) Cleavage rate (replicates = 4, cleaved embryos/putative zygotes; 0 μg/mL, 139/162; 0.5 μg/mL, 187/217; 1 μg/mL, 244/259; 5 μg/mL, 186/206; 10 μg/mL, 182/213) and (**D**) blastocyst formation rate (replicates = 4, blastocysts/putative zygotes; 0 μg/mL, 47/162; 0.5 μg/mL, 63/217; 1 μg/mL, 92/256; 5 μg/mL, 57/206; 10 μg/mL, 48/213). (**E**) The average number of total cells per blastocysts (replicates = 3, blastocysts, *n*; 0 μg/mL, *n* = 33; 0.5 μg/mL, *n* = 34; 1 μg/mL, *n* = 35; 5 μg/mL, *n* = 32; 10 μg/mL, *n* = 30). Different superscript letters (a–d) indicate statistically significant differences (*p* < 0.05).

To assess the beneficial effects of carnosine on oocyte maturation, a total of 1016 bovine COCs (replicates = 4) were exposed to various concentrations of carnosine (0 μg/mL, *n* = 210; 0.5 μg/mL, *n* = 199; 1 μg/mL, *n* = 202; 5 μg/mL, *n* = 201; 10 μg/mL, *n* = 204) for 22–24 h during IVM. As shown in Figure 1, the first polar body extrusion rate (Figure 1B) was not affected following carnosine exposure. Then, a total of 1057 COCs (replicates = 4) under different concentrations of carnosine treatment (0 μg/mL, *n* = 162; 0.5 μg/mL, *n* = 217; 1 μg/mL, *n* = 259; 5 μg/mL, *n* = 206; 10 μg/mL, *n* = 213) were used for IVF. As shown in Figure 1C,D, the proportion of presumed zygotes that were cleaved was not affected, but a significantly higher blastocyst rate was observed in the 1 μg/mL carnosine group (92/256, 36.13% ± 4.22%) compared with the control (47/162, 25.94% ± 4.62%) and the other three treatment groups (0.5 μg/mL, 63/217, 28.58% ± 4.61%; 5 μg/mL, 57/206, 27.63% ± 3.26%; 10 μg/mL, 48/213, 22.08% ± 3.84%; *p* < 0.05; Figure 1D). In addition, the average number of total cells in blastocysts (replicates = 3, blastocysts, *n*; 0 μg/mL, *n* = 33; 0.5 μg/mL, *n* = 34; 1 μg/mL, *n* = 35; 5 μg/mL, *n* = 32; 10 μg/mL, *n* = 30) derived from oocytes treated with 1 μg/mL and 5 μg/mL carnosine was notably increased compared to the control and other carnosine-treated groups (*p* < 0.05; Figure 1E).

### 3.2. Carnosine Boosts Mitochondrial Membrane Potential, Mitochondrial Activity, and ATP Content in Bovine Oocytes

**Figure 2:**
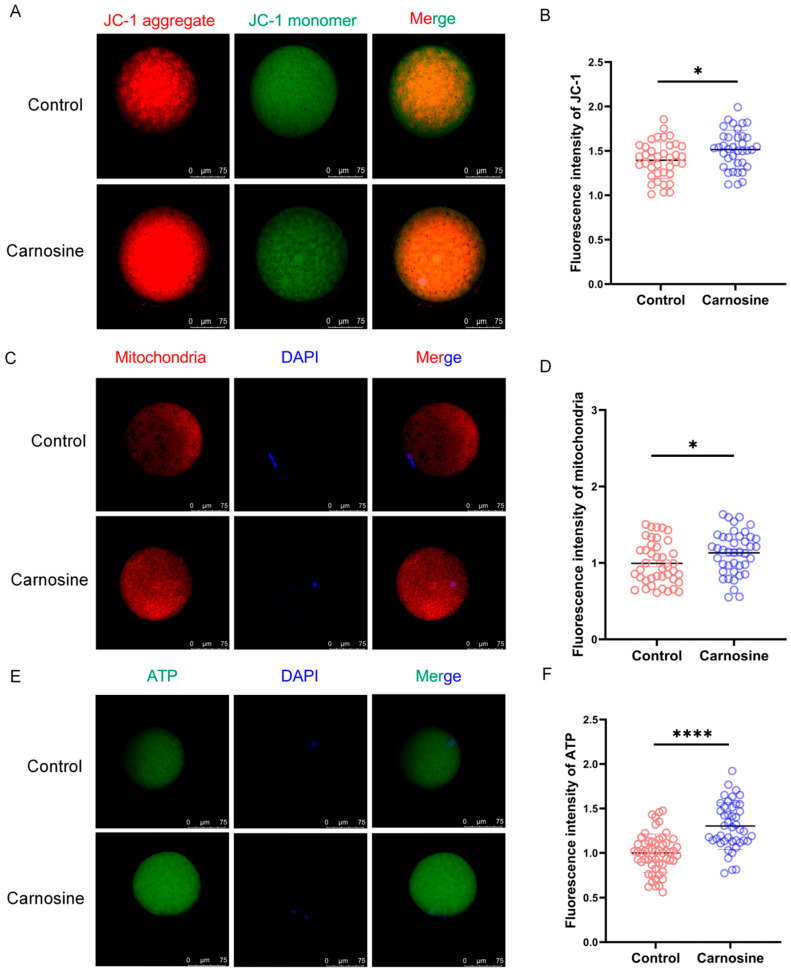
The effects of carnosine on mitochondrial membrane potential (MMP), mitochondrial activity, and ATP content. (**A**) Representative images of MMP in bovine oocytes stained with JC-1 dye. (**B**) Relative MMP expressed as the ratio of red (JC-1 aggregate; high membrane potential) to green (JC-1 monomers; low membrane potential) fluorescence intensity (replicates = 4, denuded oocytes, *n*; control, *n* = 38; carnosine, *n* = 37). (**C**) Representative images of active mitochondria in oocytes stained with MitoTracker™ Red CMXRos dye. (**D**) Relative fluorescence intensity quantification of active mitochondria (replicates = 4, denuded oocytes, *n*; control, *n* = 42; carnosine, *n* = 40). (**E**) Representative images of ATP in oocytes stained with BODIPY™ FL ATP dye. (**F**) Relative ATP fluorescence intensity quantification (replicates = 4, denuded oocytes, *n*; control, *n* = 57; carnosine, *n* = 44). Scale bar = 75 μm. * *p* < 0.05 and **** *p* < 0.0001.

The effects of carnosine on mitochondrial status in bovine oocytes were further analyzed. Significantly higher mitochondrial membrane potential was observed in carnosine-treated oocytes compared with the control group (*p* < 0.05; Figure 2A,B). Moreover, the number of active mitochondria, reflected by increased fluorescence intensity, was markedly higher in oocytes exposed to carnosine than in the control group (*p* < 0.05; Figure 2C,D). Intracellular ATP content was also elevated following carnosine treatment compared with control oocytes (*p* < 0.05; Figure 2E,F).

### 3.3. Carnosine Promotes the Antioxidant Capacity of Bovine Oocytes

**Figure 3:**
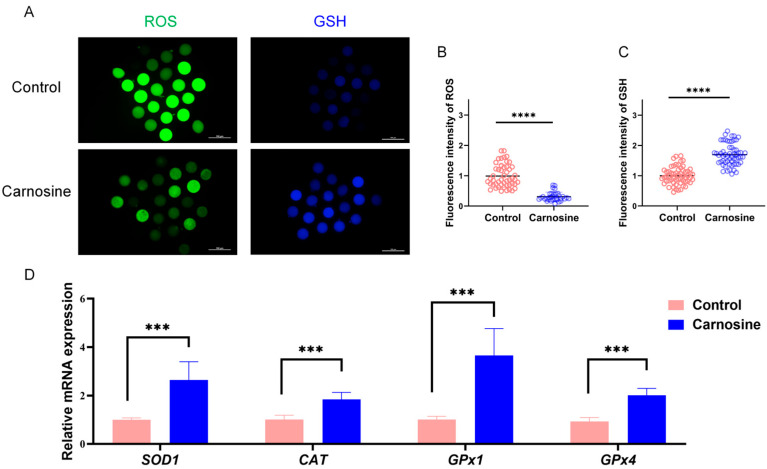
The effects of carnosine on intracellular reactive oxygen species (ROS) levels, glutathione (GSH) levels, and antioxidant gene expression. (**A**) Representative images of intracellular ROS and GSH levels in oocytes stained with 2′,7′-dichlorodihydrofluorescein diacetate (DCHFDA) and ThiolTracker™ Violet GSH detection reagent, respectively. Scale bar = 100 μm. (**B**) Relative ROS fluorescence intensity quantification (replicates = 4, denuded oocytes, *n*; control, *n* = 44; carnosine, *n* = 40). (**C**) Relative GSH fluorescence intensity quantification (replicates = 4, denuded oocytes, *n*; control, *n* = 58; carnosine, *n* = 58). (**D**) Relative mRNA levels of *SOD1*, *CAT*, *GPx1*, and *GPx4*. Three replicate experiments were performed per treatment. *** *p* < 0.001 and **** *p* < 0.0001.

To determine whether carnosine can mitigate oxidative stress in bovine oocytes, the intracellular ROS and GSH levels and the relative expression of selected antioxidant-associated genes were quantified. As presented in Figure 3, exposure of COCs to carnosine during maturation resulted in a significant decrease in intracellular ROS levels in oocytes relative to the control group (*p* < 0.05; Figure 3B). In contrast, the GSH levels were significantly higher in the carnosine treatment group than in the control group (*p* < 0.05; Figure 3C). Furthermore, the mRNA abundances of the antioxidant genes *SOD1*, *CAT*, *GPx1*, and *GPx4* were significantly upregulated following carnosine treatment (*p* < 0.05; Figure 3D).

### 3.4. Carnosine Reduces DNA Damage and Apoptosis in Bovine Oocytes

**Figure 4:**
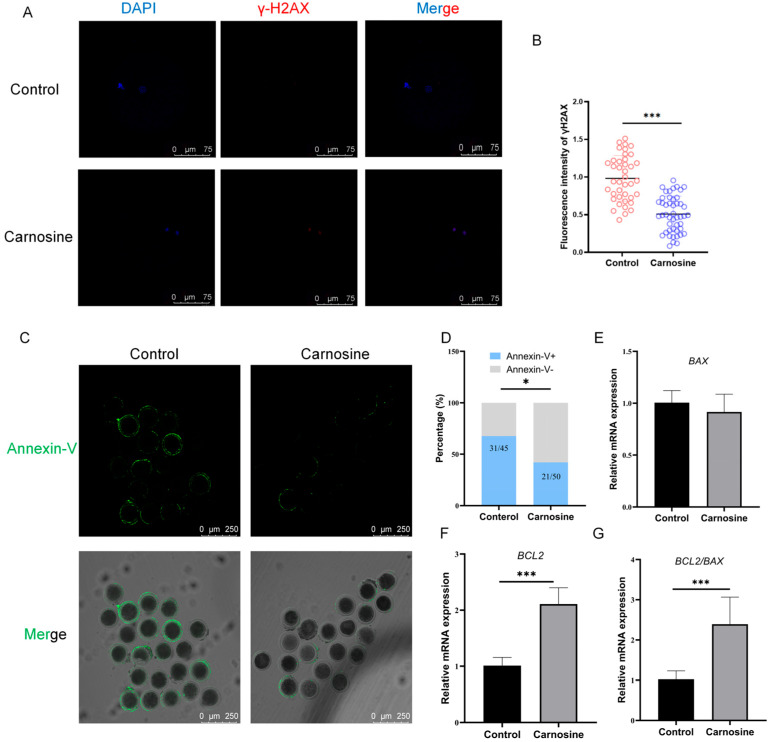
The effects of carnosine on DNA damage, early apoptosis and apoptotic-related genes expression. (**A**) Representative images of DNA double-strand break in oocytes stained with the γH2AX antibody. Scale bar = 75 μm. (**B**) Relative γH2AX fluorescence intensity quantification (replicates = 4, denuded oocytes, *n*; control, *n* = 41; carnosine, *n* = 47). (**C**) Representative images of early apoptotic oocytes stained by Annexin V-FITC. Scale bar = 250 μm. (**D**) The percentage of early apoptotic oocytes (replicates = 4, denuded oocytes, *n*; control, *n* = 45; carnosine, *n* = 50). (**E**) Relative mRNA abundance of *BAX*. (**F**) Relative mRNA abundance of *BCL2*. (**G**) The *BCL2*/*BAX* mRNA ratio. Three replicate experiments were performed per treatment. Data are presented as the mean ± SEM from three independent experiments. * *p* < 0.05 and *** *p* < 0.001.

To evaluate the efficacy of carnosine in reducing DNA damage and apoptosis in bovine oocytes, DNA double-strand breaks (DSBs), early apoptosis, and the transcripts of apoptosis-related genes were assessed. Carnosine treatment significantly decreased DSB levels, as indicated by lower γH2AX signal intensity compared with the control group (*p* < 0.05; Figure 4A,B). The Annexin-V assay showed that the early apoptotic rate in carnosine-treated oocytes was significantly lower than that in the control group (*p* < 0.05; Figure 4C,D). Although the mRNA abundance of the pro-apoptotic gene *BAX* was unaffected by carnosine exposure (*p* > 0.05; Figure 4E), the mRNA expression profile of the anti-apoptotic gene *BCL2* was markedly increased compared with the control group (*p* < 0.05; Figure 4F). Consequently, the *BCL2*/*BAX* mRNA ratio was significantly increased (*p* < 0.05; Figure 4G).

### 3.5. The Combination of Carnosine During IVM and Melatonin During IVC Synergistically Improves the Developmental Competence of Bovine Embryos

**Figure 5:**
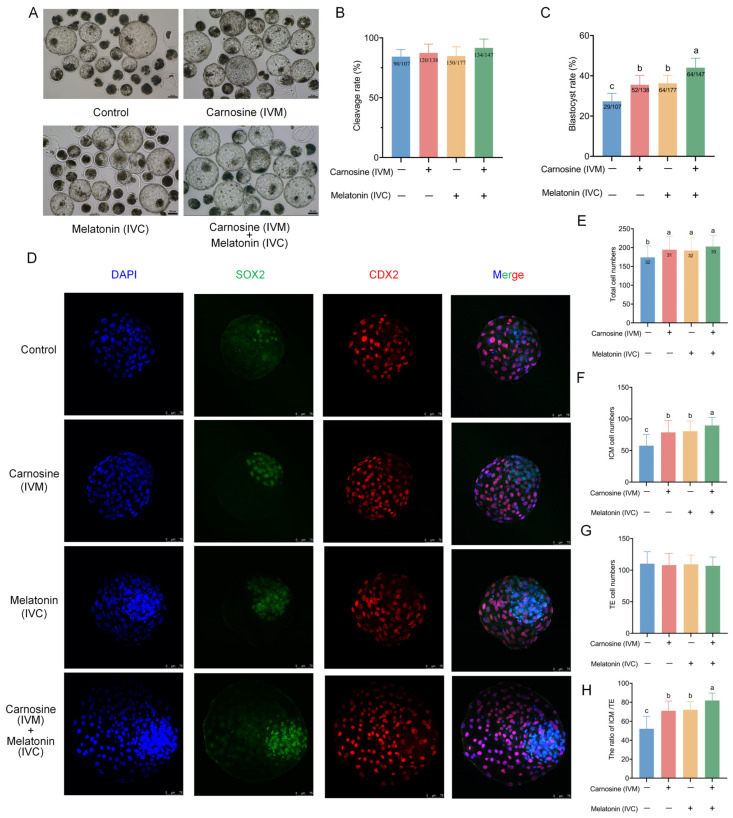
The effects of stage-specific supplementation with carnosine during in vitro maturation and melatonin during in vitro culture on the outcomes of in vitro embryo production. (**A**) Representative images of bovine day 7 embryos for each treatment group. Scale bar = 50 μm. (**B**) Cleavage rate (replicates = 4, cleaved embryos/putative zygotes; control, 90/107; carnosine (IVM), 120/138; melatonin (IVC), 150/177; carnosine (IVM) + melatonin (IVC), 134/147) and (**C**) blastocyst formation rate (replicates = 4, blastocysts/putative zygotes; control, 29/107; carnosine (IVM), 52/138; melatonin (IVC), 64/177; carnosine (IVM) + melatonin (IVC), 64/147). (**D**) Representative images of blastocyst labeling with DAPI (blue), SOX2 antibody (green), and CDX2 antibody (red). Scale bar = 75 μm. (**E**) Total cell number per blastocyst. (**F**) Inner cell mass (ICM) cell number. (**G**) Trophectoderm (TE) cell number. (**H**) The ratio of ICM/TE (replicates = 3, blastocysts, *n*; control, *n* = 32; carnosine (IVM), *n* = 31; melatonin (IVC), *n* = 32; carnosine (IVM) + melatonin (IVC), *n* = 33). Different superscript letters (a–c) indicate statistically significant differences (*p* < 0.05).

To further improve developmental efficiency, bovine oocytes were subjected to IVM with 1 μg/mL carnosine, followed by IVC of the presumptive zygotes in the presence of 10^−7^ M melatonin. No statistically significant differences in cleavage rates were observed between the treatment groups (replicates = 4, cleaved embryos/putative zygotes; carnosine (IVM), 120/138; melatonin (IVC), 150/177; carnosine (IVM) + melatonin (IVC), 134/147) and the control group (90/107; *p* > 0.05; Figure 5B). However, the synergistic treatment significantly promoted blastocyst development, yielding a dramatically higher proportion (64/147, 44.01% ± 4.74%) compared with either individual treatment group (carnosine (IVM), 52/138, 35.55% ± 4.68%; melatonin (IVC), 64/177, 36.27% ± 3.98%) or the control group (29/107, 27.33% ± 3.95%; *p* < 0.05; Figure 5C). Furthermore, the average numbers of the total and ICM cells, as well as the ICM/TE ratio of blastocysts (replicates = 3), were also significantly higher in the synergistic (*n* = 33) and individual treatment groups (carnosine (IVM), *n* = 31; melatonin (IVC), *n* = 32) compared to the control group (*n* = 32) (*p* < 0.05; Figure 5E–H), with the highest number and ratio found in the synergistic treatment group (*p* < 0.05).

## 4. Discussion

In cattle, only about one-third of oocytes matured in vitro reach the blastocyst stage, and the resulting embryos generally exhibit lower cell counts, reduced cryotolerance, and poorer post-transfer survival than their in vivo-derived counterparts [24]. Various aspects of the IVEP procedure predispose oocytes and preimplantation embryos to oxidative damage resulting from ROS overproduction [25,26]. The high lipid content and lengthy meiotic arrest render oocytes particularly susceptible to oxidative insults, which in turn damage the intracellular milieu, impair organelle function, and hinder fertilization capacity [27]. As a natural dipeptide, carnosine has garnered considerable attention as a versatile cytoprotective agent owing to its direct and indirect antioxidant activities [8]. In a comparative analysis of various antioxidants—including ascorbic acid, α-tocopherol, carnosine, imidazole-4-acetic acid, glutathione monoethylester, N-acetylcysteine, and ethoxyquin—carnosine was the only compound that reduced chromosomal breakage in Chinese hamster ovary cells under hyperoxia exposure [28]. In female rats exposed to an electromagnetic field, carnosine preserved the number and diameter of primary follicles and maintained the regular structure of the oocyte and the adjacent granulosa cells [29]. Carnosine has also been detected in seminal plasma, and shown to effectively remove lipid peroxidation products in equine samples during cooling [30]. Additionally, carnosine supplementation in cryopreservation solution promotes the recovery and maintenance of frozen-thawed bovine embryos [17]. Thus, it is plausible that carnosine may serve as a promising agent for improving reproductive outcomes. Indeed, the present results showed that supplementation of the IVM medium with 1 μg/mL carnosine yielded a higher proportion of oocytes reaching the blastocyst stage and an increased average cell number per blastocyst, highlighting the protective role of carnosine in enhancing the efficacy of IVEP in bovine.

Optimal mitochondrial function is a prerequisite for oocyte maturation, fertilization and successful early preimplantation embryo development [31]. Mitochondrial dysfunction potentially reduces ATP production, increases ROS generation, and promotes mitochondrial DNA damage, ultimately leading to compromised oocyte quality [32,33,34]. Several bioactive compounds such as melatonin [35,36,37], resveratrol [38,39], coenzyme Q10 [40,41], Mito-Q [42], and N-acetylcysteine [43] hold promise in protecting oocyte mitochondria from oxidative damage and improving their energy generation capacity. Previous studies in various experimental models have demonstrated that carnosine regulates mitochondrial function by preserving mitochondrial membrane potential, increasing the activity of respiratory chain complexes, and boosting energy production [14,44,45,46]. Consistent with these findings, the present study showed that carnosine administration during oocyte maturation resulted in higher mitochondrial membrane potential, increased mitochondrial activity and elevated intracellular ATP levels. Thus, carnosine likely ameliorates mitochondrial function, thereby enhancing subsequent fertilization and embryonic development.

A well-regulated interplay between ROS and enzymatic antioxidants (e.g., *SOD*, *CAT*, *GPx*) maintains reduced intracellular GSH levels, which is essential for healthy oocyte development [47]. Our further research revealed that supplementing the maturation medium with carnosine significantly reduced ROS levels, increased GSH contents, and upregulated the expression of *SOD1*, *CAT*, *GPx1*, and *GPx4* transcripts in bovine oocytes. This effect is most likely attributable to carnosine’s protective capacity against oxidative damage. In line with this, a previous study reported that prenatal carnosine supplementation enhanced serum antioxidant status in mice offspring, as evidenced by decreased MDA and increased SOD, GPx, and TAS levels, and concurrently benefited reflexive motor behaviors [16]. In an in vitro model of macrophage activation, carnosine not only upregulates antioxidant components (Nrf2, HO-1, and ROS scavenger enzymes) and downregulates pro-oxidant enzymes (Nox-2, Cox-2) and MDA levels but also restores energy and redox homeostasis [48]. Furthermore, accumulating evidence from ischemia–reperfusion injury studies has demonstrated that carnosine treatment effectively reduces lipid peroxidation and iron accumulation, suppresses oxidative stress, and mitigates ferroptosis and cell death [49,50]. Our findings are consistent with these outcomes, highlighting a critical role of carnosine in enhancing oocyte maturation quality.

It has been reported that hydroxyl radicals (·OH) generated via the hydrogen peroxide (H_2_O_2_)-driven Fenton reaction impair spindle formation and chromosome alignment in metaphase-II oocytes, and this damage is considered a potential contributor to poor oocyte quality [51]. The reaction between iron and H_2_O_2_, known as the Fenton reaction, produces highly toxic species (e.g., ·OH) that consequently trigger cell death [52,53,54]. As a chelator of bivalent metal ions, carnosine not only directly scavenges superoxide anion and hydroxyl radicals but also exhibits outstanding ability in blocking the Fenton reaction [8,12]. According to several reports, carnosine inhibits cell death and apoptosis by preventing ROS-induced DNA fragmentation and mitochondrial membrane potential loss, blocking the caspase cascade, and elevating the anti-apoptosis factor Bcl-2 [55,56,57,58]. In the current study, carnosine administration resulted in decreased levels of DNA DSBs, coinciding with reduced early apoptosis and increased *BCL2* mRNA abundance. Our results indicate that carnosine enhances oocyte quality through its anti-apoptotic action. Additionally, Haeri et al. reported that carnosine prevents gamma irradiation-induced testicular dysfunction via an anti-apoptotic mechanism, and this restorative effect ultimately enables spermatogenesis recovery [59]. Collectively, these findings provide compelling evidence that carnosine could serve as an effective therapeutic candidate for improving outcomes in IVEP.

To achieve higher developmental competence of oocytes and preimplantation embryos, it appears crucial to strategically apply the recognized mechanisms and favorable factors that influence in vitro procedures [60]. Melatonin, predominantly secreted by the pineal gland as a neurohormone, has been well established to participate in both male and female reproduction [61,62,63,64]. Our earlier work in bovine models revealed that melatonin not only improves oocyte and semen quality but also functions as a robust agent counteracting oxidative stress-induced insults [23,35,65]. Given the combined effects of melatonin, Amaral et al. speculated that continuous supplementation during IVEP might yield better bovine embryo outcomes than stage-specific supplementation. Unexpectedly, however, the results were inconsistent with their anticipation, as the co-administration group showed a blastocyst rate no higher—and in fact sometimes lower—than that observed with separate melatonin addition during either IVM or IVC [66]. In the present study, the impact of stage-specific supplementation with carnosine during IVM and melatonin during IVC on IVEP outcomes was assessed. As anticipated, the combined treatment synergistically promoted both the developmental potential and quality of embryos. Moreover, evidence from rat liver studies indicates that prophylactic carnosine and melatonin treatment attenuates the incidence of apoptosis, inflammation, and DNA damage triggered by titanium dioxide nanoparticles [67]. Taken together, these results suggest that carnosine and melatonin may provide complementary benefits at distinct developmental stages, and their synergistic action thereby maximizes the developmental potential of embryos.

## 5. Conclusions

**Figure 6:**
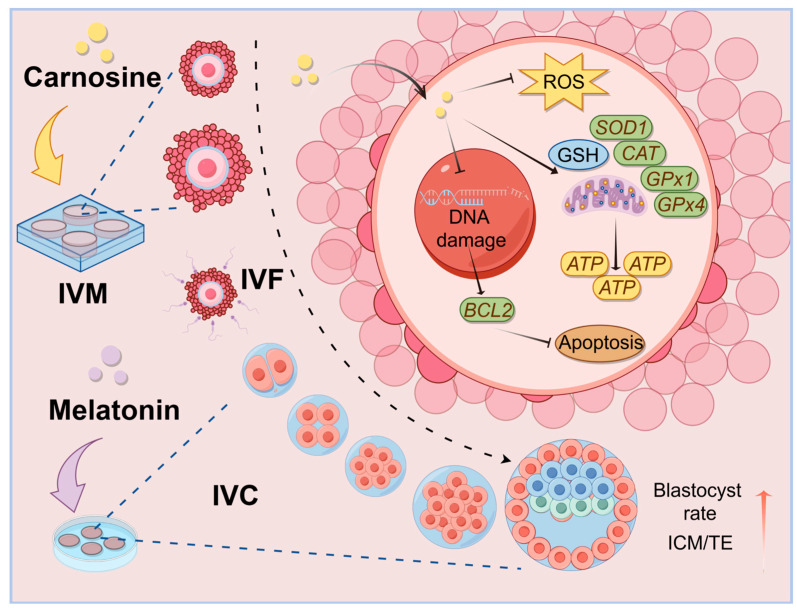
The proposed model illustrating how carnosine enhances the in vitro developmental competence of bovine oocytes. IVM, in vitro maturation; IVF, in vitro fertilization; IVC, in vitro culture; ROS, reactive oxygen species; GSH, glutathione; ICM, inner cell mass; TE, trophectoderm. Created with Figdraw.

The present study demonstrates that enriching the IVM medium with 1 μg/mL carnosine enhances bovine oocyte maturation quality and subsequent in vitro embryo development. Carnosine administration ameliorates mitochondrial function, boosts antioxidant capacity, and reduces DNA damage and early apoptosis, thereby promoting subsequent fertilization and embryonic development. Furthermore, stage-specific supplementation—carnosine during IVM and melatonin during IVC—acts synergistically to improve both blastocyst development and quality (Figure 6). These findings provide novel evidence for the therapeutic potential of carnosine in enhancing IVEP outcomes and support the adoption of stage-specific supplementation strategies to improve bovine embryo production.

## Figures and Tables

**Table 1:** Primer sequences for qRT-PCR.

Gene	Primer Sequence	Product Length (bp)	Accession Number
*β-actin*	F: GCGGCATTCACGAAACTACCTT	268	NM_173979.3
	R: TCCTGCTTGCTGATCCACATCT		
*SOD1*	F: TTCTCTACTTGGTTGGGGCG	145	NM_174615.2
	R: ACGACTGTATCTCCCTTTGCC		
*CAT*	F: GCGCAGAAACCTGATGTCCT	194	NM_001035386.2
	R: AAGTAGCCAAAAGCCCCTGC		
*GPx1*	F: CAAGAACGAGGAGATCCTGA	200	NM_174076.3
	R: GGGACCAGGTGATGAACTTA		
*GPx4*	F: GCAGAGATCAAAGAGTTCGC	104	NM_001246431.1
	R: TTCATCCATTTCCACAGAGG		
*BAX*	F: GGCTGGACATTGGACTTCCTTC	112	NM_173894.1
	R: TGGTCACTGTCTGCCATGTGG		
*BCL2*	F: TTTGTGGAGCTGTATGGC	130	NM_001166486.1
	R: TTCACTTATGGCCCAGATAG		

Note: F: forward primer; R: reverse primer.

## Data Availability

The original contributions presented in this study are included in the article. Further inquiries can be directed to the corresponding authors.
